# Insights into the Protein–Lipid Interaction of Perivitellin-2, an Unusual Snail Pore-Forming Toxin

**DOI:** 10.3390/toxins17040183

**Published:** 2025-04-06

**Authors:** Romina F. Vázquez, M. Antonieta Daza Millone, Matías L. Giglio, Tabata R. Brola, Sabina M. Maté, Horacio Heras

**Affiliations:** 1 Instituto de Investigaciones Bioquímicas de La Plata “Prof. Dr. Rodolfo R. Brenner”, INIBIOLP, CCT-La Plata, CONICET, Facultad de Ciencias Médicas, Universidad Nacional de La Plata, 60 y 120, La Plata 1900, Argentina; rvazquez@quimica.unlp.edu.ar (R.F.V.); matias.giglio@utah.edu (M.L.G.);; 2 Instituto de Investigaciones Fisicoquímicas Teóricas y Aplicadas, INIFTA, CCT-La Plata, CONICET, Universidad Nacional de La Plata, Diagonal 113 y 64, La Plata 1900, Argentina; dazamillone@inifta.unlp.edu.ar; 3 Department of Biology, University of Utah, Salt Lake City, UT 84115, USA; 4 Cátedra de Química Biológica, Facultad de Ciencias Naturales y Museo, Universidad Nacional de La Plata, 122 y 60, La Plata 1900, Argentina

**Keywords:** MACPF, snail pore-forming toxin, neurotoxin/enterotoxin, lectin, lipid membranes, protein–lipid interaction, cholesterol, brain lipids

## Abstract

The perivitellin-2 (PV2) from snails is an unusual neuro and enterotoxin comprising a pore-forming domain of the Membrane Attack Complex and Perforin Family (MACPF) linked to a lectin. While both domains have membrane binding capabilities, PV2’s mechanism of action remains unclear. We studied the apple snail *Pomacea maculata* PV2’s (PmPV2’s) interaction with lipid membranes using various biophysical and cell biology approaches. In vitro studies showed that PmPV2 toxicity decreased when cholesterol (Chol) was diminished from enterocyte cell membranes. Chol enhanced PmPV2 association with phosphatidylcholine membranes but did not induce pore formation. In contrast, using rat brain lipid models, rich in glycolipids, PmPV2 exhibited high affinity and induced vesicle permeabilization. Negative stain electron microscopy and atomic force microscopy confirmed the formation of pore-like structures in brain lipid vesicles. Our findings suggest that Chol is a necessary lipid component and point to PmPV2–glycolipid interactions as potential activators critical to triggering PmPV2’s pore-forming activity, providing insights into this novel toxin’s mechanism.

## 1. Introduction

The plasma membrane barrier is essential for cell survivorship, and disruption of its integrity results in altered cellular homeostasis and, eventually, cell death [1,2]. This is precisely how pore-forming toxins (PFTs) work: they create holes in the plasma membrane of target cells. PFTs are ubiquitously found in living organisms, from bacteria and protists to plants, fungi, and animals: they act as virulence factors of pathogenic bacteria [3], parasitic protozoans [4] and fungi [5], as humoral components of the immune system of plants and animals [6], and even as venoms to subdue their prey (e.g., sea anemones, stonefish) [7,8] or for defense against predation (apple snail eggs) [9].

PFTs are peptides and proteins usually secreted as water-soluble monomers that oligomerize, upon cell membrane recognition and binding, into ring-like or pore-like structures that eventually pierce the lipid bilayer [3,6,10]. The membrane recognition process involves specific regions of the PFT attaching to a membrane component, either a protein, a sugar moiety, or a lipid, triggering large conformational changes to insert the proteins into the membrane [11,12]. Independently of whether the PFTs bind to the target membrane through a specific lipid component or not, protein–lipid interactions are needed to penetrate the lipid bilayer and stabilize the pore structure once inserted, a process that could be strongly influenced by the membrane composition and physicochemical properties [10]. The bacterial toxins of the cholesterol-dependent cytolysin (CDC) family, for instance, need the presence of cholesterol (Chol) in the membrane to form active pores, which seems to be true for most members of the family [13]. PFT–membrane interaction is less understood in other groups, like the Membrane Attack Complex/Perforin (MACPF) family [14,15,16]. Although there is little sequence similarity, CDC and MACPF are structurally related and are considered different branches of the same superfamily, the MACPF/CDC superfamily [17]. The binding of the prototype member of the MACPF family perforin to the membrane depends not on the presence of a specific lipid but on the physical properties of the membrane: pore formation is prevented in ordered lipid phases or in the presence of negatively charged lipids [18,19]. Conversely, the fungal MACPF pleurotolysin (Ply) specifically binds to sphingomyelin and Chol-rich membranes through the PlyA chain, which in turn bind the pore-forming chain PlyB [14].

In this work, we explore the protein–membrane lipid interaction of perivitellin-2 (PmPV2), a neuro- and entero-PFT member of the MACPF family found in the poisonous eggs of two species of freshwater snails, *Pomacea maculata* Perry, 1810 (PmPV2) and *Pomacea canaliculata* (Lamark, 1828) (PcPV2) [9,20,21,22,23,24,25]. PV2 is the second most abundant protein within the eggs of these species and serves as both nutritional source for embryo development and defense against predation [20,22]. Its solution structure differs from nearly all MACPFs as instead of a monomer, it is a dimer in solution, and each of its protomers combines two immune proteins (a MACPF domain-containing protein and a lectin) into a toxin (i.e., it is a dimer of heterodimers) [23]. This binary toxin resembles AB-toxins, a group of toxins otherwise restricted to bacteria and plants [21,23]. PV2 is also unique among animals in that it has a dual function, acting as a neurotoxin causing a set of neurological signs and lethality [21,23], and also as an enterotoxin, altering the gut morphology and physiology in rodents and bullfrogs [9,24,26].

In PV2, the MACPF chain is linked by a single disulfide bond to the lectin chain, and two of these heterodimers are arranged head-to-tail by non-covalent forces in the native proteins [23]. Research on the action mechanism of this novel toxin is in its early stages, and several aspects remain unexplored. We have previously shown that the lectin chain acts as a delivery subunit targeting aminated glycans, a necessary step for pore formation by the MACPF subunit in enterocytes [23]. However, no information regarding the interaction of PV2 with membrane lipids is available.

This work addresses this issue by providing direct experimental evidence of PmPV2–membrane interactions at the lipid level, characterizing PmPV2’s interaction with synthetic and natural lipid membranes, exploring the effect of Chol using simple membrane models with varied lipid composition, and studies with intestinal cells in vitro.

## 2. Results and Discussion

### 2.1. Plasma Membrane Cholesterol Levels Modulate PmPV2 Toxicity in Gut Cells

**Figure 1:**
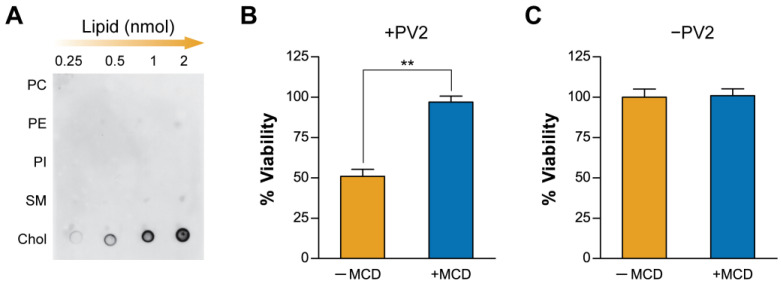
Interaction of PmPV2 with some membrane lipids. (**A**) Lipid dot blot assay of PmPV2 interaction with major membrane lipids showing affinity towards cholesterol (Chol). PC: phosphatidylcholine; PE: phosphatidylethanolamine; PI: phosphatidylinositol; SM: sphingomyelin. (**B**) Viability of Caco-2 cells treated with PmPV2 (+PV2) after reducing membrane Chol with methyl-β-cyclodextrin (+MCD, blue bars) and without MCD treatment (−MCD, orange bars), evaluated using MTT assay; ** *p* < 0.01. (**C**) Cell viability of control cells (−PV2).

To explore PV2 from *P. maculata* (PmPV2) specificity towards lipids, we first studied its interaction with immobilized lipids through a dot blot assay. As shown in Figure 1A, PmPV2 interacted with Chol in a concentration-dependent fashion, while only mild to no interaction was observed with the other plasma membrane lipid classes assayed, suggesting a relatively high affinity towards Chol. Control membranes processed without PmPV2 exhibited no non-specific antibody binding to the lipid spots (Supplementary Figure S1). Considering the literature and this result, we investigated the contribution of this lipid to the toxin activity on cultured human adenocarcinoma (Caco-2) cells after partially reducing the Chol content from their membranes. Chol-diminished cells were not affected by the toxin after 90 min incubation with PmPV2 at a concentration that decreased cell viability by over 50% in control cells (Figure 1B). No baseline effect on cell viability was detected in MCD-treated cells not exposed to PmPV2 (Figure 1C). These results suggest that PmPV2 directly interacts with Chol and that the Chol content of the membrane highly influences binding and/or pore formation. Given that many enterotoxins, including bacterial AB-toxins such as cholera toxin, exploit glycan and lipid interactions for host cell targeting, PmPV2 may function through a similar mechanism. We further explore the role of Chol in PmPV2’s action mechanism in the next sections.

### 2.2. Cholesterol Enhances PmPV2–Membrane Interaction but Is Not Essential for Toxin Association

**Figure 2:**
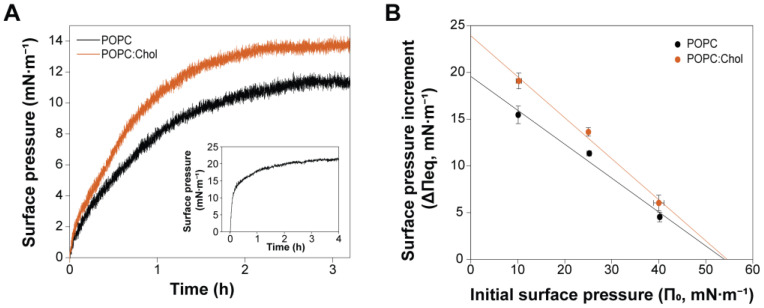
PmPV2 interaction with lipid monolayers. (**A**) Surface pressure increments (Δπ) were registered over time after injecting PmPV2 (100 nM) into the subphase buffer (HBS) beneath lipid monolayers of POPC or POPC:Chol (3:1 mole ratio) at an initial surface pressure (π_o_) of 25 mN·m^−1^. The inset shows the kinetics of PmPV2 (100 nM) adsorption to the air/buffer interface in the absence of lipids. (**B**) Total surface pressure increment (Δπ_eq_) was measured for PmPV2 (100 nM) interaction with POPC or POPC:Chol monolayers at different π_o_ values. Maximum insertion pressures (MIPs) and Δπ_o_ parameters were calculated by extrapolating the linear regression curves to the *x*- and *y*-axis, respectively. All measurements were performed at 23 ± 1 °C.

To further study the effect of Chol on the interaction of PmPV2 with membranes, we formed lipid monolayers of POPC and POPC:Chol (in 3:1 mole ratio) as simple model systems. Injecting PmPV2 into the subphase beneath the lipid monolayers increased the surface pressure (Δπ), indicating the toxin’s adsorption and incorporation into the lipid films (Figure 2). At an initial surface pressure (π_o_) of 25 mN·m^−1^, the total increment (Δπ_eq_) in POPC:Chol was 13.7 ± 0.4 mN·m^−1^ and 11.33 ± 0.07 mN·m^−1^ in neat POPC monolayers, suggesting a higher incorporation of the toxin into Chol-containing films (Figure 2A). The rate constants (k) for the association of PmPV2 with POPC:Chol were also slightly higher (1.162 ± 0.005 h^−1^) than for POPC (1.071 ± 0.005 h^−1^) monolayers. It is worth noticing that kinetics were slow, with equilibrium surface pressures typically reached in 3–4 h, a behavior similar to PmPV2 adsorption to the bare air/buffer interface (Figure 2A inset), suggesting that the lipid films did not significantly enhance PmPV2 affinity towards the interface. However, once the equilibrium was reached, PmPV2 caused higher Δπ_eq_ in Chol-containing monolayers at various initial surface pressures π_o_ (Figure 2B), indicating higher incorporation regardless of the lipid density. The average monolayer surface pressure proposed to correlate to the properties of bilayers and natural membranes is in the range of 30–35 mN·m^−1^, with fluctuations that can span this range [27,28]. The high maximum insertion pressures (MIPs) ~ 55 mN·m^−1^ calculated in both lipid systems suggest that PmPV2 could insert into biological membranes via lipid bilayer interaction. Notwithstanding, the Δπ_o_ parameter (Δπ at π_o_ = 0), which describes the monolayer capacity of modifying the surface activity of the protein [29], points to PmPV2 interacting in the interfacial (phosphate) region with POPC monolayers—giving similar Δπ_o_ values to those produced by the adsorption of PmPV2 to the air/buffer interface (inset in Figure 2A)—while results in Chol-containing films suggest a deeper insertion into this system.

Altogether, our monolayer assays indicate that while Chol is not required for PmPV2 association, it enhances the toxin interaction with the membrane.

### 2.3. PmPV2 Does Not Permeabilize POPC/Chol Vesicles but Induces Content Release with Strong Affinity in Brain Lipid Models

**Figure 3:**
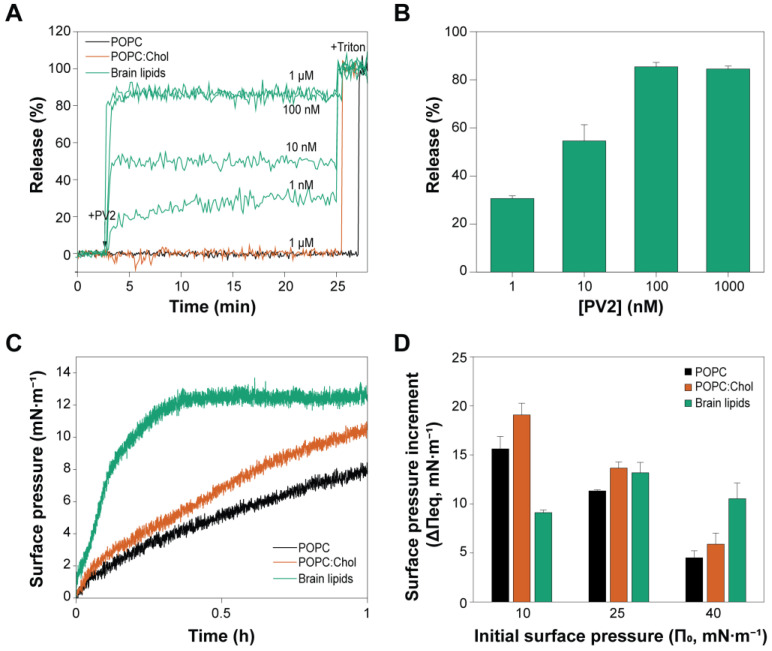
PmPV2 activity in models containing brain lipids. (**A**) Time course of ANTS efflux from unilamellar vesicles (100 µM lipids) made of POPC, POPC:Chol (in 3:1 mole ratio), or brain lipids in the presence of the indicated concentrations of PmPV2. The arrow depicts the time PmPV2 was added to the vesicle suspensions. The total fluorescence corresponding to 100% release was obtained after vesicle disruption by adding Triton X-100. (**B**) Percent released at different PmPV2 concentrations in vesicles made of brain lipids. Measurements were performed at 20 ± 1 °C and are reported as mean ± SD, N = 3. (**C**) Surface pressure increment (Δπ) vs. time curves obtained for the interaction of PmPV2 (100 nM) with brain lipid monolayers at an initial surface pressure of 25 mN·m^−1^ (green trace). (**D**) Total surface pressure increment registered at equilibrium (Δπ_eq_) for the interaction of PmPV2 (100 nM) with brain lipid monolayers (green bars) at different initial surface pressures (π_o_). Results reported for POPC (black) and POPC:Chol 3:1 (red) (Figure 2) were included for comparison.

We tested whether Chol would enhance the pore-forming capacity of PmPV2 by analyzing the release of the aqueous content from POPC or POPC:Chol vesicles (Figure 3A). Surprisingly, no release activity was detected in either type of lipid vesicle, even at high toxin concentrations (protein–lipid ratios ranging from 1:10^5^ to 1:10^2^). Measurements were conducted over 18 h, and even at this large time lapse, no membrane permeabilization was detected. Given the neurotoxic properties of PmPV2 [21], we tested vesicles made from rat brain lipids. These brain lipid vesicles displayed a concentration-dependent release of their contents (Figure 3A,B). At 1 nM PmPV2, the release was 30.4 ± 0.8% and increased to a maximum of 85% at 100 nM or higher concentrations.

These results prompted us to explore the insertion of PmPV2 into brain lipid monolayers. As shown in Figure 3C, brain lipids drastically enhanced the association rates of PmPV2 (with the rate constant k decreasing from ~1.1 h^−1^ in POPC and POPC:Chol to 0.0900 ± 0.0005 h^−1^ in brain lipids at an initial surface pressure π_o_ of 25 mN·m^−1^). This indicates a stronger affinity of PmPV2 for brain lipids. However, the total surface pressure increments produced by the toxin were only higher in brain lipids compared to POPC or POPC:Chol monolayers at high initial surface pressures (40 mN·m^−1^), i.e., more condensed lipid packing (Figure 3D). In POPC and POPC:Chol, the typical Δπ_eq_ vs. π_o_ behavior was observed: as the initial surface pressures π_o_ increased, the Δπ_eq_ decreased since the tighter lipid packing restricted protein insertion. In contrast, brain monolayers showed lower Δπ_eq_ at 10 mN·m^−1^ compared to 25 mN·m^−1^. This behavior is indeed striking since it indicates greater PmPV2 incorporation in more densely packed lipid systems. This surprising behavior may be explained by the need for concentration or clustering of a specific component present in the brain lipid films.

The whole brain lipids contain a complex lipid repertoire. This remarkably enhanced the affinity and insertion of PmPV2 into the monolayers and induced the permeabilization of vesicles, indicating that the lipid composition highly influences PmPV2 interaction with the membrane and that specific lipids may be required to trigger pore formation. Previous results showed that the lectin domain is involved in the pore-formation process by PmPV2 on Caco-2 cells [23]. Considering that besides PC and Chol, the brain is particularly enriched in glycosphingolipids [30], these could act as initial binding sites for PmPV2 through its lectin domain. Interestingly, lectins have multivalent binding sites and are usually associated with promoting the clustering of receptors at the cell surface [31]. Monolayers at 10 mN·m^−1^ represent a system with low lipid packing and higher molecular distances between the lipid components. The low effects observed at this π_o_ may stem from the need for nearby lipid receptors to potentiate the interaction of PmPV2. More work is needed to explore this issue.

### 2.4. Brain Lipids Promote an Irreversible Association of PmPV2 with Lipid Bilayers

**Figure 4:**
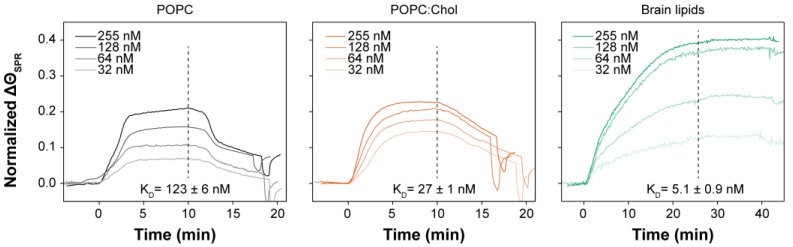
PmPV2 interaction with supported lipid bilayers. Sensorgrams showing the interaction of different concentrations of PmPV2 (32–255 nM) with supported bilayers composed of POPC, POPC:Chol (3:1 mole ratio), or brain lipids. PmPV2 was injected at the depicted concentrations (t = 0) at 10 µL/min. The dashed lines indicate the time at which PmPV2 injection ended. The change in minimum SPR angle (∆Θ_SPR_) was normalized considering the amount of immobilized lipids before each binding assay. K_D_ values were obtained from ∆Θ_SPR_ at 11 min for POPC and POPC:Chol and 25 min for brain lipids for each toxin concentration (N = 4).

We used SPR to follow the real-time interaction of PmPV2 with supported lipid bilayers (SLBs), providing insights into its affinity for different membrane systems. SLBs were formed by fusing lipid vesicles of neat POPC, POPC:Chol (in 3:1 mole ratio), or brain lipids on the surface of the SPR sensor chips. Figure 4 shows the sensorgrams obtained for each lipid composition after PmPV2 injection at 10 µL/min. Higher normalized signal intensities (∆Θ_SPR_) were obtained in the brain lipid system, pointing to the binding of a larger amount of protein, while more time was needed to reach a steady state signal. This kinetic contrasted the fast association to brain lipids observed in the monolayer assays (Figure 3C) and could arise from the whole process from binding to pore-formation occurring in the presence of brain lipids, as suggested by the permeabilization experiments (Figure 3A,B). In addition, no significant dissociation was detected, evidencing an irreversible association of PmPV2 with the brain bilayers. The calculated K_D_ values were 123 ± 6 nM, 27 ± 1 nM, and 5.1 ± 0.9 nM, for the interaction with POPC, POPC:Chol, and brain lipid SLBs, respectively, confirming a higher affinity for the brain lipid system. Comparing POPC and POPC:Chol SLBs, a higher membrane affinity and slower dissociation kinetics were found in the presence of Chol. The pronounced drop in the SPR signal after the association step in POPC SLBs reflected weak PmPV2–membrane interactions without Chol. Reducing the Chol content from 3:1 to 9:1 resulted in a slight decrease in PmPV2 membrane affinity (K_D_ = 32.9 nM), but still higher than for the POPC bilayers.

As a whole, results from SPR measurements confirmed a higher affinity and strong association of PmPV2 with brain lipids. Also, they reinforced the monolayer assay results suggesting that Chol favors the interaction with the membrane.

### 2.5. Brain Lipids Drive PmPV2 Pore Formation

**Figure 5:**
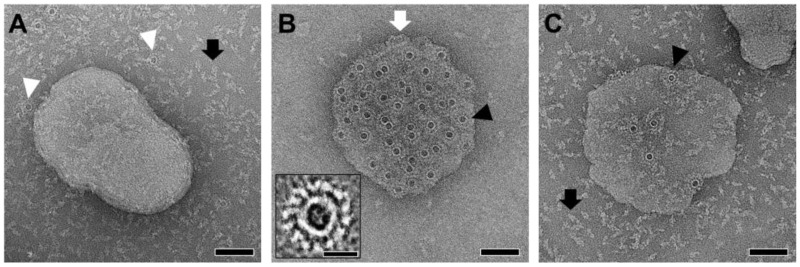
TEM imaging of PmPV2 pores formed on lipid vesicles. Three types of vesicles were incubated with PmPV2: (**A**) POPC:Chol vesicles (3:1 mole ratio). (**B**) Vesicles made of 100% brain lipids. (**C**) Vesicles containing a 1:1 mixture of brain lipids and POPC: Chol (50% brain lipids). PmPV2 oligomerized in pore-like structures only in vesicles containing brain lipids (**B**,**C**) while it remained mostly soluble in POPC:Chol ones (black arrow in **A**,**C**). Inset in (**B**) shows the rosette-like structure of PmPV2 assemblies (bar 10 nm). White arrowheads in (**A**) mark ring-like structures formed on the carbon grid surface. White arrow: side view; black arrowheads: top view of ring-like structures. Bar 50 nm.

To further explore pore formation in lipid membranes, which has been suggested in leakage experiments, PmPV2 was incubated with vesicles either made of POPC:Chol 3:1, brain lipids, or a combination of the two. These vesicles were then imaged using negative staining transmission electron microscopy (TEM) (Figure 5). In line with the results from the release experiments (Figure 3), when PmPV2 was incubated with POPC:Chol vesicles, it remained mostly soluble, and no pore-like structures were observed on the liposome surface while a few ring-like structures were detected on the carbon grid. These results contrasted with a previous study in which pore-like structures were presumably detected in PC:Chol liposomes by TEM [23]. CDC toxins assemble into transient pre-pores on the membrane surface, while for MACPFs, this intermediate state is extremely rare [32]. It could be that the interaction of PmPV2 with hydrophobic surfaces triggered the formation of pre-pores of a size similar to membrane pores in that study, as seen on the carbon grid surface in Figure 5A. In turn, vesicles containing brain lipids showed clearly visible pore assemblies inserted in the lipid bilayer (Figure 5B). When using a mixture of synthetic POPC:Chol and brain lipids (1:1), the number of pores in the membrane decreased significantly, and the protein remained mostly soluble (Figure 5C). These results further confirm that a specific lipid component and/or lipid environment present in brain–lipid membrane models is essential for PmPV2 pore formation and toxic activity. Modifying the lipid composition of the brain system, even with physiologically relevant lipids already present in the brain—like PC and Chol [33]—drastically reduced the number of pores, highlighting the strong dependence of PmPV2 on a specific environment. Mixing brain lipids with PC:Chol involves a reorganization of lipids, decreased concentration of other specific brain lipid components, and changes in the membrane properties. These could account for the lower pore-forming activity found in this mixture. Along with the reduced effect observed in monolayers at low lipid density (Figure 3D), these results indicate that the lipid content and distribution on the model membranes also modulate the pore-forming activity of PmPV2. This finding aligns with the reported dependence of lectin activity on the glycolipid–phospholipid ratio [34,35], supporting the idea that the PmPV2 lectin domain is involved in protein–membrane interaction and the pore-formation process in brain lipids. While our findings suggest a potential pre-pore stage (Figure 5A), further studies are needed to confirm this hypothesis.

While the TEM resolution did not allow for precise characterization of the pore-like assemblies’ structure and monomer count, we estimated inner and outer diameters to be 7.8 ± 0.4 and 20.6 ± 1.0 nm (N = 60), respectively, and 14–15 monomers forming the ring (N = 20). These structures differed widely from the large transmembrane pores of CDC toxins, comprising 30 to 50 monomers with inner diameters of 25 to 30 nm [36,37], and also from typical MACPF perforin assemblies, which contain more monomers (18–30) and form larger pores than PmPV2, with inner diameters of 10–20 nm [38]. The TEM micrographs suggest that PmPV2 pores are more defined, resembling only those of MACPF protein pleurotolysin B, which forms rosette-like structures of 12–14 subunits with an inner diameter of ~8 nm [14]. Interestingly, pleurotolysin B acts in an AB toxin array, requiring a second protein to recognize and guide it to the target membrane for pore formation [39,40]. PmPV2 itself is a MACPF-lectin AB toxin [23]. Our results support the idea that the interaction of the lectin domain with a still uncharacterized brain glycolipid may be necessary for the proper protein rearrangements that lead to pore formation by the MACPF domain. This is evidenced in the lack of oligomeric assemblies and permeabilization activity in simple membrane models like PC:Chol bilayers, a commonly used model in which other members of the MACPF/CDC family, including perforin and perfringolysin O, form active pores [36,41].

### 2.6. AFM Imaging of PmPV2 Pores Suggests Lectin–Glycolipid Interactions at the Bilayer Surface

**Figure 6:**
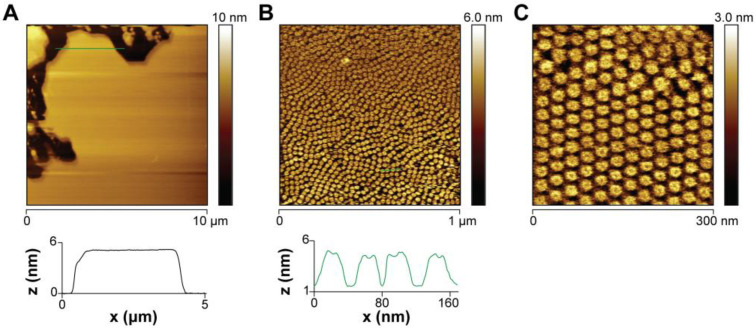
Atomic force microscopy imaging of PmPV2 pores in brain bilayers. (**A**) Topographic image (10 µm × 10 µm) and line profile of a supported brain lipid bilayer before PmPV2 addition. (**B**) AFM topography 15 min after the addition of PmPV2 (50 nM) to the supported brain bilayer (1 µm × 1 µm) and line profiles of protruding pores. (**C**) Topographic image of a smaller area (300 nm × 300 nm).

The pores formed by PmPV2 were then studied under more physiological conditions using AFM imaging on SLBs made from brain lipids in a liquid environment. The topographical images before PmPV2 treatment showed a smooth, homogeneous bilayer surface on the brain SLBs, with no detectable phase segregation (Figure 6A). However, 15 min after adding PmPV2, the bilayer was almost completely covered by pore-like assemblies densely packed in hexagonal arrays (Figure 6B,C). The particle analysis estimated an external diameter of 21 ± 1 nm (N = 120), consistent with TEM results, and an average pore height from the bilayer surface of 3.8 ± 0.3 nm. The mean height of these structures was lower than those reported for other MACPF/CDCs toxins, which extend 10 nm above the membrane for perforin or pleurotolysin B and 7 nm for CDC proteins [14,37,42,43]. Notwithstanding, the pore-forming lectin CEL III from the sea cucumber *Cucumaria echinata* form flat rings ~4 nm in height after the lectin domain binds membrane surface glycans and its pore-forming domains oligomerize [44,45]. The flat ring contacting the surface glycans was proposed to stabilize the transmembrane beta-barrel pore [44]. PmPV2 is a MACPF lectin and the differences in pore height compared to other MACPF/CDC family members could derive from this particular architecture. The fact that tip convolution effects might be underestimating the pore heights should also be considered [46].

Toxins from the CDC family are known to interact with membrane Chol to form functional pores. The exact role of Chol in the pore formation process of CDC toxins largely varies and does not explain their cellular specificity per se [47]. Many CDCs also have high-affinity interactions with glycans, suggesting that glycoproteins and/or glycolipid receptors contribute to their cell specificity and toxic activity [48,49,50,51]. Our findings support this hypothesis for the MACPF PmPV2, suggesting that lectin–glycolipid interactions may determine its cytotoxicity. Even when PmPV2 interacted with Chol in the lipid-overlay assay, its pore-forming and permeabilization activities were not observed in simple models containing only Chol and PC. However, these activities readily occurred when lipids came from rat brains. This suggests that specific lipid components in the brain are crucial for PmPV2 oligomerization and membrane disruption. Glycolipids are abundant in the outer leaflet of brain membranes [30,52] and may play a role in PmPV2 lectin–glycan interactions, facilitating binding, concentration, and proper protein organization necessary for transmembrane pore formation and neurotoxic effects [21]. In addition to neurotoxicity, PmPV2 also exhibits enterotoxic activity [9], confirmed here by its cytotoxicity towards Caco-2 cells. Glycolipids are known targets of other enterotoxic proteins [53,54] and could serve as binding sites for PmPV2 in enterocytes. It is worth noting that while PmPV2 is not hemolitically active, it causes agglutination of erythrocytes [21], reflecting that specific toxin–glycolipid/glycoprotein interactions may be required for the sequential steps to form the pore. PmPV2 activity is inhibited by selected monosaccharides, further suggesting that lectin recognition is essential to trigger PmPV2 pore formation in enterocytes [23]. Recent studies report that Chol and glycans bind independently to the PFT Streptolysin, Pneumolysin, and Listeriolysin [51]. Our results showed that PmPV2 presents higher affinity and selective activity in brain lipids and also that Chol enhances PmPV2’s interaction and has a stabilizing effect in the toxin–membrane interaction, particularly decreasing the detachment from the lipid bilayer. Similarly, the CDC Intermedilysin (Ily) binds to PC:Chol liposomes but lacks pore-forming activity in this system [55]. Unlike other CDCs that are activated to oligomerize by Chol, Ily activation needs binding to a membrane glycoprotein. Notwithstanding, Chol–protein interactions are necessary to maintain the Ily pre-pore complex attached to the membrane for its consequent insertion into the lipid bilayer [56]. Likewise, PmPV2 showed enhanced interactions with the model membranes in the presence of Chol, and its toxic effect decreased in Chol-depleted Caco-2 cells, indicating that PmPV2-Chol interactions play a role in forming and stabilizing the pore assemblies. Ongoing research will address this topic.

## 3. Conclusions

The pore-forming toxins produced by certain organisms target the host cells through the interaction with specific lipids usually absent in the toxin-producing organisms. Here, we have characterized the interaction of the snail MACPF/CDC toxin PmPV2 with Chol, demonstrating that while PmPV2 interacts favorably with this sterol, other lipid membrane components are necessary for successful pore formation. Given that Chol comprises 86–90% of the total sterol content in snails [57], the other lipid requirements reported here for PmPV2 are likely to protect snail membranes from its toxic activity. Our results suggest this is achieved by PmPV2’s dual-domain architecture—unique in animals, making PmPV2 an evolutionary and functional outlier—that enables PmPV2 to recognize glycolipid components of exogenous membranes. The rosette-like pore assemblies found using brain lipid systems point to PmPV2–glycolipid interactions as potential activators for pore formation. The findings from this work open new avenues for studying this novel pore-forming toxin, including its interaction with specific lipid components, the influence of Chol in pore dynamics, and the structural characterization of the pores formed by PmPV2. Future research will focus on these aspects to further understand PmPV2′s mechanism of action, unique among animals, together with its potential applications in biomedicine.

## 4. Materials and Methods

### 4.1. Reagents and Materials

1-Palmitoyl-2-oleoyl-glycero-3-phosphocholine (POPC) and cholesterol (Chol) were purchased from Avanti Polar Lipids (Birmingham, AL, USA). DL-dithiothreitol (DTT), methyl-β-cyclodextrin (MCD), N-(2-Hydroxyethyl)piperazine-N′-(2-ethanesulfonic acid) (HEPES), NaCl, and other reagents were purchased from Sigma Aldrich (St. Louis, MO, USA). Chloroform and methanol, HPLC-grade, were purchased from Merck (Darmstadt, Germany). Gold sensor slides, glass substrates coated with a 50 nm gold layer (SPR102-AU), were obtained from Bionavis (Tampere, Finland) and muscovite mica grade V-1 was purchased from SPI supplies (West Chester, PA, USA). AFM tips were purchased from Bruker (model DNP-10, Camarillo, CA, USA). The ultrapure Milli Q water (Merck Millipore, Burlington, WI, USA) used for all the solutions and experiments had a resistivity of 18.2 MΩ.cm at 23 °C.

### 4.2. Protein Purification

PmPV2 was purified from fresh-laid *P. maculata* egg clutches as previously reported [23,58] and diluted in PBS buffer (1.5 mM NaH_2_PO_4_, 8.1 mM Na_2_HPO_4_, 140 mM NaCl, 2.7 mM KCl, pH 7.4). Total protein was quantified from its molar extinction coefficient [19] using an Agilent 8453 UV/Vis diode array spectrophotometer (Agilent Technologies, Waldbronn, Germany).

### 4.3. Brain Lipids Extraction

Lipids were extracted from the brains of Wistar rats using Folch’s method with modifications [59]. Briefly, brain tissue was homogenized in a cold buffer containing protease inhibitors, followed by the addition of 20 volumes of chloroform–methanol (2:1, *v*/*v*). The homogenate was then filtered, and water was added to the filtrate at 20% of the total volume. The mixture was vortexed again and then allowed to stand at 4 °C until phase separation. The lower phase, containing the brain lipid extract, was collected. To further purify the lipid extract and remove residual polar contaminants, the lower phase was washed twice with a solvent mixture resembling the upper phase composition (chloroform–water–methanol, 3:48:47, *v*/*v*). Lipids were resuspended in chloroform and stored at −80 °C.

### 4.4. Lipid Dot Blot

One microliter of each lipid class was spotted onto nitrocellulose membranes (GE Healthcare, Amersham, UK) from a 1 mM stock chloroform solution. Two equivalent membranes were prepared. Membranes were dried at room temperature for 1 h, and then blocked for 2 h in 3% (*w*/*v*) non-fat dried skimmed milk in TBS-10 buffer (10 mM Tris-HCl, 150 mM NaCl, pH 7.4). Membranes were incubated with either TBS-10 buffer (negative control) or a solution of 67 nM PmPV2 in TBS-10 buffer for 3 h at room temperature on an orbital shaker. Membranes were washed 5 times in TBS-10 buffer for 5 min and then incubated with rabbit anti-serum against PmPV2 (1:1000 dilution) in 3% (*w*/*v*) non-fat milk in TBS-10 buffer for 1 h. Next, the membranes were washed as described above and incubated with secondary goat anti rabbit-IgG antibodies conjugated with horseradish peroxidase (BIO-RAD, Hercules, CA, USA) (1: 4000 dilution) for 2 h. Finally, membranes were washed again as above and developed by chemiluminescence. The presence of lipid dots was confirmed by incubation of the membrane in an iodine chamber.

### 4.5. Influence of Membrane Cholesterol on Cytotoxicity

PmPV2 cytotoxicity was evaluated using the Caco-2 cell line obtained from ATCC (Cedarlane Inc., Burlington, ON, USA) as previously described [9,21]. In brief, cells were seeded in 200 mL of Dulbecco’s modified Eagle medium (DMEM) on 48-well plates at densities that ensured approximately 90% confluence after 24 h. Cells were used with and without Chol depletion pretreatment to evaluate the effect of Chol on PmPV2 cytotoxicity. Membrane Chol was diminished by pretreatment with 5 mM methyl-β-cyclodextrin (MCD) in DMEM for 1 h at 37 °C, which removes from 18.3 to 27.3% of membrane Chol [60]. After MCD pretreatment, 50 μL/well of PmPV2 in PBS were added (0.11 mg/mL final concentration) and incubated at 37 °C for 90 min. Fifty microliters of PBS were used in control cells. Cell viability was determined by the 3-(4,5-dimethythiazol-2-yl)-2,5-diphenyl tetrazolium bromide (MTT) assay [61], measuring the absorbance at 560 nm using a microplate Multimode Detector DTX-880 (Beckman Coulter, Inc., Brea, CA, USA). Cell viability was expressed as control percentage: Viability (%) = (OD treated cells/OD control cells) × 100.

### 4.6. Liposome Preparation

Multilamellar vesicles were prepared by mixing the appropriate volumes of synthetic POPC, Chol, or brain lipids dissolved in HPLC-grade chloroform–methanol (2:1, *v*/*v*). Samples were dried by evaporating the solvent under a stream of nitrogen and then with high vacuum for 2 h. The samples were hydrated in a desired volume of HEPES buffered-saline (HBS, 20 mM HEPES, 150 mM NaCl, pH 7.4) at 30 °C for POPC and POPC:Chol and at 70 °C for brain lipids, with stirring to facilitate dispersion. Multilayered vesicles were sonicated in an FB-15049 bath sonicator (Fisher Scientific Inc., Waltham, MA, USA) at 30 °C (POPC and POPC:Chol) or 70 °C (brain lipids) for 1 h to obtain small unilamellar vesicles (SUVs) for surface plasmon resonance (SPR) and atomic force microscopy (AFM) experiments or extruded through polycarbonate-filters (Avestin, Inc., Ottawa, ON, Canada) to generate large unilamellar vesicles (LUVs).

### 4.7. Interaction with Lipid Monolayers

Surface pressure measurements were performed with a NIMA Langmuir trough Model 102M (NIMA Technology, Coventry, UK) by the Wilhelmy method using HBS Buffer as subphase. The lipid monolayers were formed by spreading dropwise the POPC, POPC:Chol (3:1 mole ratio), or brain lipids solutions prepared in chloroform–methanol (4:1, *v*/*v*) onto the subphase surface until the desired initial surface pressure (π_o_) was attained. After being spread, the monolayers were left for 10 min to equilibrate and allow complete solvent evaporation. PmPV2 from a stock solution prepared in HBS Buffer was then injected with a Hamilton microsyringe into the subphase bulk to achieve a final concentration of 100 nM. After toxin injection, the increment in surface pressure against time (Δπ vs. t) was recorded until a stable signal was obtained. At this point, the total increment in surface pressure (Δπ_eq_) was determined. The maximum insertion pressure (MIP) of the protein in POPC and POPC:Chol monolayers was determined from the linear regression curves of the corresponding Δπ_eq_ vs. π_o_ plots by extrapolating the curves to Δπ_eq_ = 0.

The kinetics of insertion of the toxin into the lipid monolayers at initial surface pressures of 25 mN·m^−1^ were analyzed by fitting the Δπ vs. t plots according to Equation (1), from which the rate constant (k) for the insertion of PmPV2 into the different films was obtained [62].

Δπ = Δπ_eq_ (1 − e^−kt^) (1)

The surface-active properties of the protein were assessed using the same experimental setup by analyzing its adsorption kinetics in the absence of lipids. The data were analyzed using SigmaPlot 12.0 (Systat Software, San Jose, CA, USA). All the experiments were performed at 23 ± 1 °C in triplicate and are reported as mean ± SD values.

### 4.8. Permeabilization Assays

Large unilamellar vesicles composed of POPC, POPC:Chol 3:1, or brain lipids were used to study the ability of PmPV2 to form active pores in these model membranes. Release of the vesicle’s aqueous content was measured by the 8-aminonaphthalene-1,3,6-trisulfonic-acid/N,N′-p-xylene-bis-pyridinium-bromide (ANTS/DPX) assay [63]. Vesicles of the different lipid compositions containing 12.5 mM ANTS, 45 mM DPX, 20 mM Tris- HCl, 70 mM NaCl, pH 7.4 were prepared by ten cycles of freeze–thawing and subsequent extrusion of the lipid suspension through two stacked polycarbonate-filters with 100 nm pore diameter (Avestin, Inc., Ottawa, ON, Canada). The untrapped material was removed by gel filtration on Sephadex G-75 using 20 mM Tris- HCl, 150 mM NaCl, pH 7.4 (TBS) as the elution Buffer. The lipid concentration of the eluted vesicles was determined by measuring the phospholipid fraction by the phosphorus colorimetric method [64], considering the phospholipid to total lipids ratio in each sample.

PmPV2 was added at different final concentrations to the vesicle suspensions (100 µM lipid concentration), and the increment in fluorescence (F) of ANTS was evaluated as indicative of membrane permeabilization. Fluorescence measurements were performed at 20 ± 1 °C using a SLM 4800 Aminco spectrofluorometer (SLM Instruments, Inc., Urbana, IL, USA) at excitation and emission wavelengths of 355 nm and 530 nm, respectively.

The fluorescence intensity corresponding to 100% release was determined by the addition of 5 µL of 10% (*v*/*v*) Triton X-100 to the vesicle suspension (0.25% final concentration). Release (%R) was calculated as follows:

%R= 100 (F_t_ − F_o_)/(F_TX-100_ − F_o_) (2)

where F_t_ is the fluorescence intensity at time t after the addition of the toxin, and F_o_ and F_TX-100_ are the fluorescence intensities before protein and after Triton X-100 addition, respectively. Measurements were performed in triplicate and are reported as mean ± SD.

### 4.9. Surface Plasmon Resonance (SPR) Measurements

SPR measurements were performed in Kretschmann configuration using a BioNavis multiparametric SPR Navi™ 220 (MP-SPR) device (Tampere, Finland) equipped with two independent lasers (670 and 785 nm) in a dual channel system. Measurements were performed in angular-scan mode (59–72 degrees), recording SPR curves every 3.5 s at a constant temperature of 22 °C. SPR gold substrates were ex situ modified with a self-assembled DTT monolayer as described in Daza Millone et al. [65]. Then, the DTT-gold substrates were placed in the SPR flow chamber and washed with HBS + 10 mM CaCl_2_ buffer (500 μL/min) and 1% Triton X-100 aqueous solution (1 min at 50 μL/min) before bilayer immobilization. The vesicle suspensions (0.2 mg/mL) were injected at 10 μL/min for 10 min for each specified composition (POPC:Chol 1:0, 9:1, and 3:1) and 15 min for brain lipids. Unbound vesicles were washed with HBS + 10 mM CaCl_2_ buffer (500 μL/min), and the amount of immobilized lipids was recorded after 5–10 min of signal stabilization. For binding assays, PmPV2 at different concentrations from 32 to 255 nM in HBS Buffer was injected for 10–25 min at 10 µL/min on a fresh bilayer prepared for each binding assay (measurements were made at least by quadruplicate). For each measurement, a fresh DTT-gold surface was used, and a new bilayer generated.

Dissociation constant (K_D_) calculation: the overall change in minimum SPR angle (∆Θ_SPR_) was normalized considering the amount of immobilized lipids (∆Θ~ 0.4°) before each binding assay. Individual curves from t = 0 until 10–15 min after the end of the injection period were analyzed by means of TraceDrawer 1.5 software (Uppsala, Sweden). Each curve was fitted with a one-to-one depletion corrected model in the kinetic evaluation program. Mean velocity constants (k_a_ and k_d_) were obtained, and mean K_D_ calculated for each system.

### 4.10. Negative-Stain Transmission Electron Microscopy (NS-TEM)

PmPV2 pores assembled in lipid membranes were studied by TEM at LNNano-CNPEM facilities, Brazil (proposal ID 20210379). Large unilamellar vesicles (LUVs) were prepared following the method described previously. In short, three different LUV compositions were assayed: (1) a mixture of synthetic POPC and Chol (3:1 mole ratio); (2) lipids from rat brain, extracted by the method of Folch [59]; (3) a mixture of POPC:Chol + brain lipids (mixed in 1:1 ratio). Lipid mixtures were dried under a stream of nitrogen and then with a high vacuum for 1.3 h in a Speed-vac prior to TEM experiments. Lipids were then hydrated with buffer (20 mM TRIS-HCl, pH 7.4) to a final concentration of 3 mM, resuspended as multilamellar vesicles, and extruded to form LUVs. For TEM experiments, 50 µL of each LUV suspension was incubated with 2.1 μM PmPV2 for 4 min at room temperature. For NS assays, 5 μL of each solution was placed onto negatively glow discharged carbon-coated grids. After 1 min, the grids were washed with distilled water, blotted by filter papers, and stained with 2% (*w*/*v*) aqueous uranyl acetate for 15 s. The remaining liquid was removed with filter paper. Micrographs were taken on a Talos Arctica (Thermo Fisher Scientific, Waltham, MA, USA) operated at 200 kV with a FEI Falcon III detector.

### 4.11. Atomic Force Microscopy (AFM)

Freshly cleaved mica was pretreated with 120 μL of HBS Buffer containing 3 mM CaCl_2_ for 15 min at 25 °C. Small unilamellar vesicles of brain lipids (65 μL, 150 μM) were then added on top of the mica and left to adsorb and extend for 30 min at 65 °C. The samples were left for a further 60 min to equilibrate at room temperature and then washed 10 times with HBS Buffer to remove the unadsorbed vesicles. A small amount of buffer was always left on top of the mica to keep the supported bilayers hydrated during the whole process. The mica was placed in a liquid cell, and the lipid bilayers were left to equilibrate for another 30 min before measurements. Brain SLBs were imaged before and after the addition of 50 nM PmPV2 to the fluid cell.

AFM measurements were performed on a multimode atomic-force microscope controlled by a Nanoscope-V unit (Veeco Instruments Inc., Santa Barbara, CA, USA). V-shaped Si_3_N_4_ probes (DNP-10, Bruker, Camarillo, CA, USA) with spring constants of 0.06–0.18 N/m were used in contact and tapping mode. All the measurements were performed at a temperature of 23 °C. Resolution images of 512 × 512 pixels were collected at a scanning rate between 0.5 and 1 Hz. The height and error signal (vertical deflection) images were taken simultaneously. Images were analyzed using Gwyddion v2.50 software [66].

## Data Availability

The original contributions presented in this study are included in this article and supplementary material. Further inquiries can be directed to the corresponding authors.

## Supplementary Materials

The following supporting information can be downloaded at: https://www.mdpi.com/article/10.3390/toxins17040183/s1, Figure S1: Control lipid dot blot without incubating with PmPV2.

## Abbreviations

The following abbreviations are used in this manuscript:

AFM	Atomic Force Microscopy
ANTS	8-aminonaphthalene-1,3,6-trisulfonic-acid
CDC	cholesterol-dependent cytolysins
Chol	cholesterol
DPX	N,N′-p-xylene-bis-pyridinium-bromide
HBS	HEPES buffered saline
LUVs	large unilamellar vesicles
MACPF	membrane attack complex and perforin family
MCD	methyl-β-cyclodextrin
MIP	maximum insertion pressure
MLVs	multilamellar vesicles
NS-TEM	Negative-stain Transmission Electron Microscopy
PFT	pore-forming toxins
PC	phosphatidylcholine
PE	phosphatidylethanolamine
PI	phosphatidylinositol
POPC	1-palmitoyl-2-oleoyl-glycero-3-phosphocholine
PmPV2	Perivitellin-2
SLBs	supported lipid bilayers
SM	sphingomyelin
SPR	Surface Plasmon Resonance
SUVs	small unilamellar vesicles
TBS	Tris-buffered saline
