# Supplementary figures and images for: Insights into the Protein–Lipid Interaction of Perivitellin-2, an Unusual Snail Pore-Forming Toxin
Source: Toxins (Basel). 2025 Apr 6;17(4):183. doi: 10.3390/toxins17040183 (PMC12031178; doi:10.3390/toxins17040183)

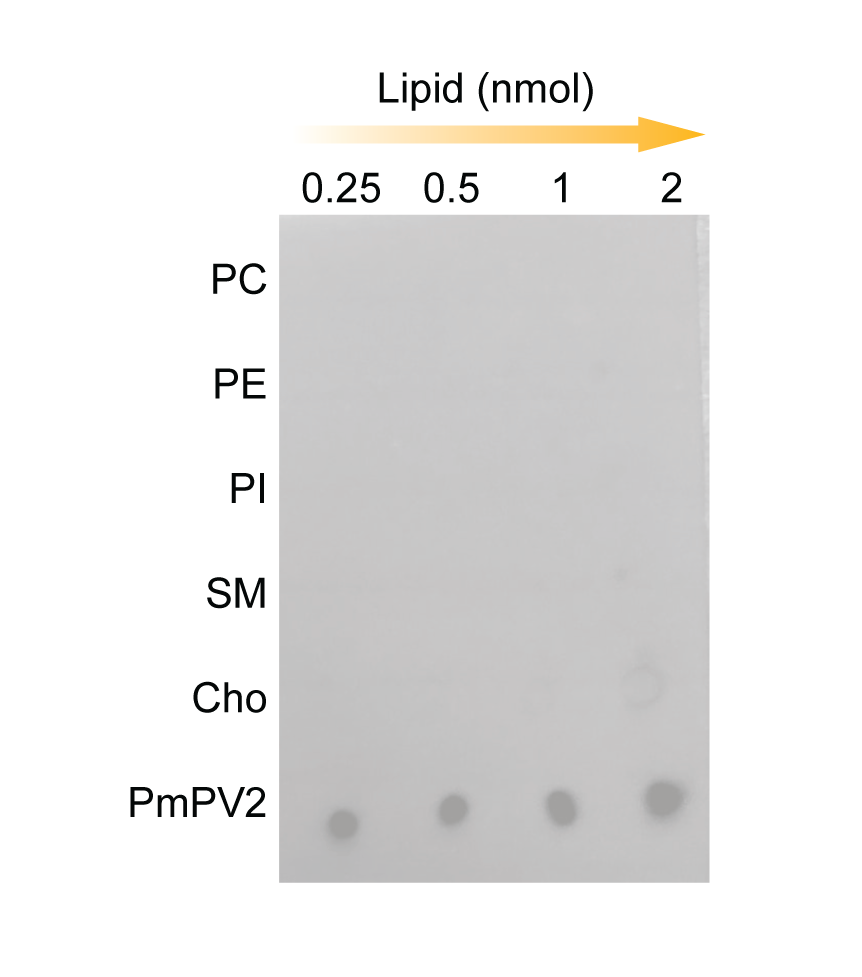

Supplement: Supplementary file 1 [file toxins-17-00183-s001.zip › toxins-3533612-supplementary.tif]
